# Insurance Status and Quality of Care in Infective Endocarditis: A National Analysis of Disparities in Length of Stay, Discharge, and Mortality

**DOI:** 10.3390/jcm15124738

**Published:** 2026-06-18

**Authors:** Joseph Hozayen, Omar Hozayen, Benjamin J. Behers, Nicolas Riveros, Anas Abu Jad, Bashar Roumia, Christoph A. Stephenson-Moe, Matthew W. Miller, Karen M. Hamad

**Affiliations:** Internal Medicine Residency Program, Sarasota Memorial Health System, College of Medicine, Florida State University, Sarasota, FL 34239, USAmatthew-miller@smh.com (M.W.M.); karen-hamad@smh.com (K.M.H.)

**Keywords:** infective endocarditis, healthcare disparities, insurance status, length of stay, outpatient parenteral antimicrobial therapy

## Abstract

**Background**: Infective endocarditis (IE) requires 4–6 weeks of intravenous antimicrobial therapy, and timely transition to outpatient parenteral antimicrobial therapy (OPAT) allows clinically stable patients to complete treatment outside the hospital. Because OPAT requires home infusion services or post-acute facility placement that typically depend on coverage, insurance status may strongly influence length of stay (LOS); national data on this association in IE remain limited. **Methods**: We performed a retrospective cross-sectional analysis of the 2016–2019 National Inpatient Sample (NIS) using ICD-10-CM codes I33 and I38 to identify adult IE hospitalizations. Patients were classified as insured (Medicare, Medicaid, or private insurance) or uninsured (self-pay or no charge). Outcomes included mean and prolonged LOS (>14 and >28 days), in-hospital mortality, discharge against medical advice (AMA), and hospitalization costs. Comparisons used chi-square and Student’s *t*-tests with appropriate NIS survey weighting. Multivariable Gamma regression (LOS, cost) and logistic regression (binary outcomes) were performed, adjusting for age, sex, race/ethnicity, income quartile, injection drug use (IDU), Elixhauser Comorbidity Index, and hospital characteristics, with an insurance × IDU interaction term. **Results**: Of 87,211 weighted IE hospitalizations, 81,667 (93.6%) were insured and 5544 (6.4%) were uninsured. Uninsured patients were younger (mean age 40.1 vs. 59.4 years) with lower comorbidity burden but higher injection drug use (IDU) prevalence (38.7% vs. 15.5%). Mean LOS was longer among the uninsured (15.5 vs. 12.4 days, *p* < 0.001); LOS > 14 days occurred in 35.8% vs. 26.6%, and LOS > 28 days in 18.5% vs. 9.2% (both *p* < 0.001). AMA discharge was four-fold higher among the uninsured (22.2% vs. 5.5%, *p* < 0.001), while unadjusted in-hospital mortality was similar (9.0% vs. 9.4%, *p* = 0.32). LOS and AMA disparities persisted in both IDU and non-IDU subgroups, with a six-fold AMA disparity among non-IDU patients (15.2% vs. 2.5%). Based on multivariable analysis, uninsured status remained independently associated with prolonged LOS > 28 days (adjusted odds ratio [aOR] 1.46, 95% CI 1.30–1.65), AMA discharge (aOR 3.51, 95% CI 3.10–3.97), and—after accounting for age and comorbidity differences—higher in-hospital mortality (aOR 1.25, 95% CI 1.10–1.43). **Conclusions**: Uninsured adults hospitalized with IE experienced longer stays, markedly higher AMA rates, and—after adjustment for age and comorbidity—higher in-hospital mortality than insured patients. These findings are consistent with nonclinical barriers to discharge—particularly limited OPAT and post-acute care access—and suggest that the younger, less comorbid profile of uninsured patients masks an underlying outcome disparity. The results identify uninsured IE patients as a population that may benefit from alternative care models and policy reforms expanding safe post-acute antimicrobial therapy.

## 1. Introduction

Infective endocarditis (IE) is a severe infection of the heart valves that carries substantial morbidity and mortality, with reported in-hospital mortality of 15–25% and one-year mortality approaching 40% [1,2,3,4,5,6,7]. Management of IE typically requires 4 to 6 weeks of intravenous antimicrobial therapy—among the longest antibiotic courses for any infectious disease [2,3]. This prolonged treatment duration creates unique challenges for healthcare delivery and discharge planning, with implications that extend well beyond the immediate medical care of the patient.

Outpatient parenteral antimicrobial therapy (OPAT) has emerged as the standard approach for completing prolonged intravenous antibiotic courses outside the hospital [8]. OPAT enables appropriately selected patients to receive their remaining antibiotic therapy at home or in a skilled nursing facility (SNF), reducing length of stay and overall healthcare costs [9,10,11,12]. Beyond economic benefit, OPAT allows patients to return to their homes, families, and communities—preserving employment, maintaining social connections, and restoring autonomy during a prolonged illness. Multiple studies have demonstrated the safety and efficacy of OPAT for IE, with outcomes comparable to those achieved with full inpatient treatment [9,10,11,12,13,14,15,16]. Current guidelines from the Infectious Diseases Society of America and the American Heart Association endorse OPAT for stable IE patients meeting specific clinical and social criteria [2,8].

Access to OPAT, however, is not uniform. A critical but often underappreciated determinant of OPAT eligibility is health insurance status. OPAT requires substantial infrastructure—home health nursing, infusion supplies, laboratory monitoring, and often SNF placement—services that typically depend on insurance coverage or substantial out-of-pocket resources [17,18]. Patients without adequate coverage may face insurmountable barriers to OPAT access, potentially resulting in prolonged hospitalization solely for completion of antibiotic therapy that could otherwise be safely delivered in outpatient settings [17]. For these patients, what should be a period of recovery at home becomes weeks of institutional confinement. Complicating matters further, antimicrobial resistance in IE—particularly among organisms such as methicillin-resistant *Staphylococcus aureus* and enterococci—may necessitate prolonged or alternative antibiotic regimens that further extend the required duration of intravenous therapy and compound barriers to timely discharge.

The intersection of IE management and insurance status represents an important but understudied area of healthcare disparities. While prior research has documented insurance-related disparities for various cardiovascular outcomes [19,20,21,22,23,24], national data specifically examining how insurance status influences length of stay in IE remain limited [25]. Quantifying these disparities is essential for informing policy discussion, resource allocation, and the development of alternative care models that may improve access to appropriate post-acute care for underserved populations.

In this study, we used the National Inpatient Sample (NIS), the largest publicly available all-payer inpatient database in the United States [26,27], to examine the association between insurance status and length of stay in patients with IE. We hypothesized that uninsured patients would experience significantly longer hospital stays than insured patients, reflecting barriers to discharge rather than differences in disease severity. Our specific aims were to (i) describe LOS and prolonged-LOS rates by insurance status; (ii) describe in-hospital mortality, discharge disposition, and cost by insurance status; (iii) determine whether observed disparities were attributable to differences in injection drug use (IDU) prevalence by performing prespecified subgroup analyses; and (iv) evaluate the independence of observed associations through multivariable regression adjustment.

## 2. Materials and Methods

### 2.1. Study Design and Reporting

We conducted a retrospective cross-sectional analysis of the National Inpatient Sample (NIS) for the years 2016–2019. The study followed the Strengthening the Reporting of Observational Studies in Epidemiology (STROBE) guideline for cross-sectional studies. The analytic plan was prespecified and descriptive: the primary aim was to characterize the association between insurance status and hospital length of stay in adults hospitalized with IE.

### 2.2. Data Source

The NIS is the largest publicly available all-payer inpatient healthcare database in the United States, developed as part of the Healthcare Cost and Utilization Project (HCUP) and sponsored by the Agency for Healthcare Research and Quality [26]. The database contains data from approximately 7 million unweighted hospital stays per year, representing a 20% stratified sample of all discharges from community hospitals nationwide; when appropriately weighted, it yields nationally representative estimates of approximately 35 million hospitalizations annually. Variables include patient demographics, principal and secondary diagnoses, procedures, hospital characteristics, length of stay, charges, and discharge disposition. Because the NIS contains only de-identified, publicly available data, this study was deemed exempt from institutional review board review (see Institutional Review Board Statement).

### 2.3. Study Population

We identified adult hospitalizations (age ≥ 18 years) with a principal or secondary diagnosis of IE using ICD-10-CM codes I33 (acute and subacute endocarditis) and I38 (endocarditis, valve unspecified). These codes have been validated in prior administrative database studies of IE with positive predictive values exceeding 80% [28,29]. Hospitalizations were excluded if the primary expected payer was missing or coded as “other,” because heterogeneity within this category would have prevented meaningful interpretation.

### 2.4. Exposure Variable

The primary exposure was insurance status, derived from the primary expected payer variable in the NIS and dichotomized as insured or uninsured. Insured patients included those with Medicare (traditional Medicare and Medicare Advantage), Medicaid, or private insurance (commercial plans and health maintenance organizations). Uninsured patients included those with self-pay or no charge as the expected payer. We chose binary classification a priori for two reasons: (i) the principal mechanistic hypothesis—that absence of any third-party coverage limits OPAT and SNF access—is binary in structure; and (ii) prior NIS studies of insurance disparities have used this classification, facilitating comparability. Insurance status reflects the primary expected payer at the time of discharge; the implications of in-hospital coverage changes are addressed in Section 4.5.

### 2.5. Outcome Variables

The primary outcome was length of stay (LOS), analyzed as a continuous variable (mean and median) and as two clinically motivated dichotomous variables: LOS > 14 days, corresponding to the inflection point at which OPAT-eligible patients would typically have been discharged; and LOS > 28 days, corresponding to extreme prolonged hospitalization spanning the full duration of a typical IE antibiotic course. Secondary outcomes were in-hospital mortality, discharge against medical advice (AMA), routine discharge home, and total hospitalization costs. Costs were estimated by multiplying total charges by hospital- and year-specific cost-to-charge ratios provided by HCUP, as charges do not accurately reflect actual resource utilization.

### 2.6. Covariates

Covariates included age, sex, self-reported race/ethnicity (White, Black, Hispanic, and other—as recorded in the NIS), median household income quartile based on patient ZIP code, hospital characteristics (bed size, teaching status, urban/rural location, geographic region), and comorbidity burden assessed using the Elixhauser Comorbidity Index [30]. We separately identified clinically relevant conditions including diabetes mellitus, chronic kidney disease, heart failure, injection drug use (IDU), HIV/AIDS, prior valve surgery, and cardiac surgery during the index hospitalization. Race/ethnicity is reported as a social construct that may capture exposure to structural racism rather than a biological variable.

### 2.7. Statistical Analysis

Descriptive statistics characterized baseline demographics, clinical features, and outcomes by insurance status. Categorical variables are reported as weighted percentages and compared using Pearson chi-square tests; continuous variables are reported as means with standard deviations or medians with interquartile ranges (IQR) and compared using Student’s *t*-tests. To examine whether observed disparities were attributable to differential IDU prevalence between insurance groups, we performed prespecified subgroup analyses among IDU and non-IDU patients.

To evaluate the independence of observed associations, we performed multivariable regression analyses. LOS (continuous) and total hospitalization cost were modeled using Gamma regression with a log link to accommodate positive, right-skewed distributions. Binary outcomes (LOS > 14 days, LOS > 28 days, AMA discharge, in-hospital mortality) were modeled using logistic regression; results are reported as adjusted odds ratios (aOR) with 95% confidence intervals. All models adjusted for age, sex, race/ethnicity, median household income quartile, injection drug use, Elixhauser Comorbidity Index score (with the drug abuse component excluded to avoid double-counting with the IDU variable), hospital bed size, hospital teaching status, and hospital region. An insurance × IDU interaction term was included to formally test for effect modification. Insurance status was coded as a binary variable (uninsured [self-pay or no charge] vs. insured [Medicare, Medicaid, or private]; insured as reference).

All analyses incorporated NIS survey design elements (discharge weight, hospital cluster) to produce nationally representative estimates with valid variance estimation, in accordance with HCUP analytic guidance [27]. Hospitalizations with missing values for the exposure or primary outcome were excluded; missingness for covariates was <2% and was handled by complete-case analysis. Statistical significance was defined as a two-sided *p* < 0.05. Descriptive analyses were performed in Stata 17 (StataCorp, College Station, TX, USA); multivariable models were estimated in Python 3.13.1 using the statsmodels package version 0.14.4 with frequency weights and cluster-robust standard errors.

## 3. Results

### 3.1. Study Cohort

Between 2016 and 2019, we identified 87,211 weighted hospitalizations for IE among adults that met inclusion criteria. Of these, 81,667 (93.6%) had insurance coverage (Medicare, Medicaid, or private) and 5544 (6.4%) were uninsured (self-pay or no charge).

### 3.2. Baseline Characteristics

Baseline characteristics stratified by insurance status are presented in Table 1. Uninsured patients were substantially younger than insured patients (mean age 40.1 vs. 59.4 years, *p* < 0.001). Sex distribution was similar between groups (54.5% vs. 55.7% male, *p* = 0.09). Uninsured patients were more likely to be White (75.3% vs. 70.6%) and Hispanic (10.0% vs. 8.1%), and less likely to be Black (9.1% vs. 12.8%) (*p* < 0.001 for the overall distribution). Uninsured patients were more likely to reside in ZIP codes within the lowest median household income quartile (43.4% vs. 31.8%, *p* < 0.001).

Clinical characteristics differed markedly between groups in a pattern consistent with the age differential. Uninsured patients had significantly higher rates of injection drug use (38.7% vs. 15.5%, *p* < 0.001). Conversely, insured patients had substantially higher prevalence of age-related comorbidities: diabetes mellitus (31.0% vs. 12.8%, *p* < 0.001), chronic kidney disease (31.4% vs. 9.1%, *p* < 0.001), and heart failure (42.2% vs. 20.6%, *p* < 0.001). HIV prevalence was low in both groups but slightly higher among uninsured patients (1.9% vs. 1.4%, *p* = 0.003). Insured patients were more likely to undergo cardiac surgery during the index hospitalization (44.0% vs. 39.7%, *p* < 0.001).

### 3.3. Length of Stay

Uninsured patients experienced substantially longer hospitalizations than insured patients (Table 2). Mean LOS was 15.5 ± 17.4 days among uninsured patients compared with 12.4 ± 14.8 days among insured patients, a difference of 3.1 days (*p* < 0.001). Median LOS was also longer among uninsured patients (9.0 days [IQR 4.0–22.0] vs. 8.0 days [IQR 4.0–15.0], *p* < 0.001), with the wider interquartile range in uninsured patients reflecting a subset with markedly prolonged stays.

**Figure 1:**
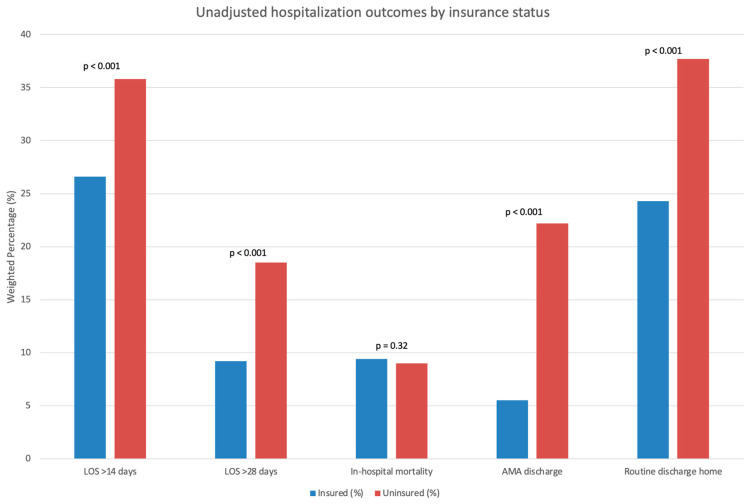
Unadjusted weighted percentages for key hospitalization outcomes by insurance status. Insured *n* = 81,667; uninsured *n* = 5544. *p*-values from Pearson chi-square tests.

The disparity was more pronounced when examining prolonged hospitalization. Among uninsured patients, 35.8% had LOS exceeding 14 days compared with 26.6% of insured patients (*p* < 0.001), a 35% relative increase. The disparity widened further at the 28-day threshold: 18.5% of uninsured patients experienced LOS > 28 days compared with 9.2% of insured patients (*p* < 0.001), representing a two-fold increase in the risk of extreme prolonged hospitalization among uninsured patients (Figure 1).

### 3.4. Mortality, Discharge Disposition, and Cost Outcomes

Despite the substantial differences in LOS, in-hospital mortality was similar between groups: 9.0% among uninsured patients compared with 9.4% among insured patients (*p* = 0.32). The lack of a mortality difference, despite longer stays among uninsured patients and despite their younger age and lower comorbidity burden, suggests that the prolonged hospitalizations are not driven by greater disease severity or clinical complexity in the uninsured population.

Discharge disposition differed markedly between groups. Uninsured patients were more likely to be discharged home routinely (37.7% vs. 24.3%, *p* < 0.001), likely reflecting limited access to post-acute care facilities. Strikingly, uninsured patients were four times more likely to leave against medical advice (AMA): 22.2% compared with 5.5% among insured patients (*p* < 0.001). This high AMA rate suggests that some uninsured patients, facing prolonged hospitalization without a clear discharge pathway, ultimately choose to leave before completing their antibiotic course.

Despite longer LOS, mean hospitalization costs were lower among uninsured patients (USD 41,061 vs. USD 44,393, *p* = 0.02). This paradox likely reflects differences in case-mix, with insured patients having higher rates of comorbidities, higher rates of cardiac surgery (44.0% vs. 39.7%), and potentially more intensive care utilization.

### 3.5. Stratified Analysis by Injection Drug Use Status

To determine whether the observed disparities were driven primarily by the IDU population, we performed prespecified subgroup analyses comparing insured and uninsured patients within IDU and non-IDU strata (Table 3). LOS disparities persisted in both subgroups. Among non-IDU patients, uninsured individuals had longer mean LOS (14.4 vs. 11.8 days), higher rates of prolonged hospitalization > 14 days (33.1% vs. 24.5%), and doubled rates of extreme prolonged hospitalization > 28 days (15.3% vs. 7.5%) compared with insured non-IDU patients. Among IDU patients, uninsured individuals similarly demonstrated longer stays (17.2 vs. 16.0 days mean LOS) and higher rates of extreme prolonged hospitalization (23.5% vs. 18.1% for >28 days).

The AMA discharge disparity was particularly striking in the non-IDU subgroup. Among non-IDU patients, uninsured individuals had a six-fold higher rate of AMA discharge compared with insured patients (15.2% vs. 2.5%). In the IDU subgroup, AMA rates were elevated in both insurance categories but remained significantly higher among uninsured patients (33.4% vs. 22.4%). These findings demonstrate that insurance-related disparities in LOS and AMA discharge are not solely attributable to the IDU population [31,32,33,34,35,36] but represent a consistent pattern across patient subgroups.

**Figure 2:**
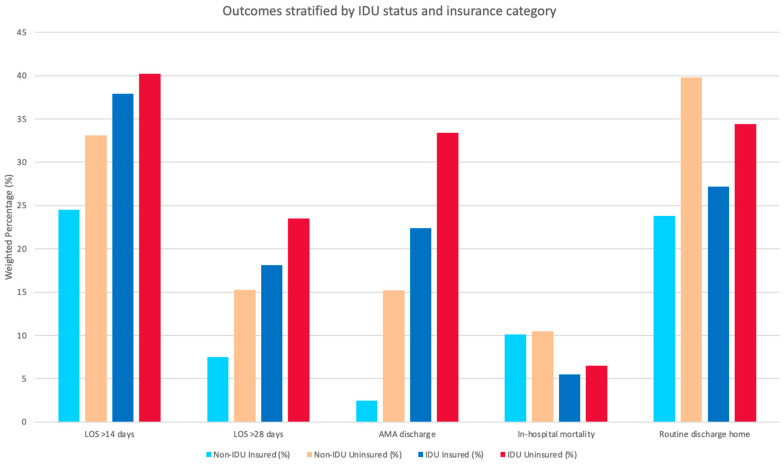
Hospitalization outcomes stratified by injection drug use (IDU) status and insurance category. Light shades indicate non-IDU subgroup; dark shades indicate IDU subgroup. Blue indicates insured patients; red indicates uninsured patients. LOS = length of stay; AMA = against medical advice.

In-hospital mortality patterns differed by IDU status. IDU patients had lower overall mortality (5.5–6.5%) than non-IDU patients (10.1–10.5%), consistent with the younger age and predominantly right-sided endocarditis in the IDU population. Within each IDU stratum, mortality was similar between insured and uninsured patients, further supporting the interpretation that LOS disparities are driven by discharge barriers rather than disease severity (Figure 2).

### 3.6. Multivariable Analysis

On multivariable Gamma regression adjusting for age, sex, race/ethnicity, income quartile, IDU, Elixhauser Comorbidity Index, and hospital characteristics, uninsured status remained independently associated with longer LOS (exponentiated coefficient 1.09, 95% CI 1.04–1.14, *p* < 0.001), corresponding to an approximately 9% longer adjusted length of stay compared with insured patients. Logistic regression confirmed significantly higher adjusted odds of prolonged hospitalization among uninsured patients: aOR 1.20 (95% CI 1.10–1.31, *p* < 0.001) for LOS > 14 days and aOR 1.46 (95% CI 1.30–1.65, *p* < 0.001) for LOS > 28 days.

AMA discharge showed the largest adjusted effect: uninsured patients had 3.5-fold higher adjusted odds of leaving against medical advice (aOR 3.51, 95% CI 3.10–3.97, *p* < 0.001). In-hospital mortality, which was similar between groups in the unadjusted analysis (9.0% vs. 9.4%, *p* = 0.32), was significantly higher among uninsured patients after adjustment for age and comorbidity burden (aOR 1.25, 95% CI 1.10–1.43, *p* < 0.001). This finding indicates that the unadjusted equivalence in mortality reflected the counterbalancing effects of a younger, less comorbid case-mix among uninsured patients. Total hospitalization costs were approximately 10% lower among uninsured patients after adjustment (exponentiated coefficient 0.90, 95% CI 0.83–0.98, *p* = 0.018).

The insurance × IDU interaction term was statistically significant for AMA discharge (interaction aOR 0.44, *p* < 0.001) and LOS > 14 days (interaction aOR 0.85, *p* = 0.019), and borderline for LOS > 28 days (interaction aOR 0.86, *p* = 0.079). The significant AMA interaction indicates that the insurance–AMA association was stronger among non-IDU patients than among IDU patients, consistent with the six-fold unadjusted AMA disparity observed in the non-IDU subgroup (Table 3, Panel A). The interaction was not significant for in-hospital mortality (*p* = 0.50) or continuous LOS (*p* = 0.85). Adjusted estimates for all outcomes are summarized in Table 4.

**Table 4:** Multivariable-adjusted associations between insurance status and hospitalization outcomes.

Outcome	Adjusted Estimate	95% CI	*p*-Value	Interactionp (ins × IDU)
**Length of stay**				
LOS, days (IRR ^†^)	1.09	1.04–1.14	<0.001	0.85
LOS > 14 days (aOR)	1.20	1.10–1.31	<0.001	0.019
LOS > 28 days (aOR)	1.46	1.30–1.65	<0.001	0.079
**Discharge**				
Discharge against medical advice (aOR)	3.51	3.10–3.97	<0.001	<0.001
**Mortality and cost**				
In-hospital mortality (aOR)	1.25	1.10–1.43	<0.001	0.50
Total cost ^‡^ (exp. coeff.)	0.90	0.83–0.98	0.018	—

**Figure 3:**
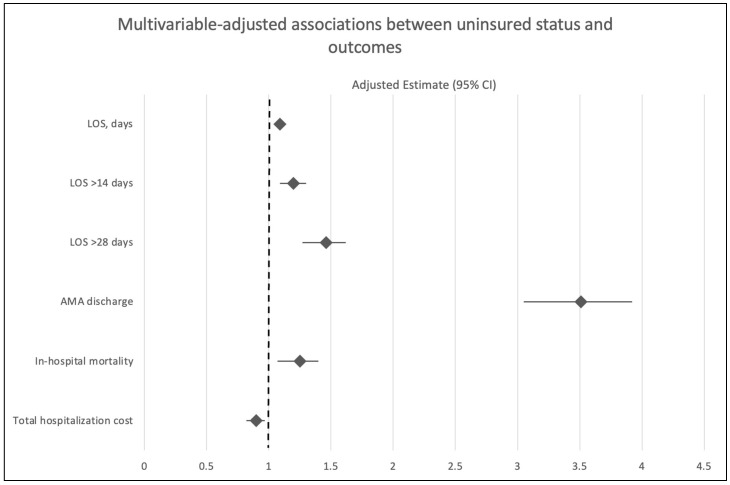
Multivariable-adjusted associations between uninsured status and hospitalization outcomes in infective endocarditis. Point estimates (diamonds) and 95% confidence intervals (horizontal lines) from frequency-weighted Gamma regression (LOS, cost) and logistic regression (binary outcomes), adjusted for age, sex, race/ethnicity, income quartile, IDU, modified Elixhauser score, and hospital characteristics. The vertical dashed line indicates the null value (estimate = 1.0). aOR = adjusted odds ratio; IRR = incidence rate ratio.

All models adjusted for age, sex, race/ethnicity, median household income quartile, injection drug use (IDU), Elixhauser Comorbidity Index (modified), hospital bed size, teaching status, and region. Analyses used frequency-weighted Gamma GLM (LOS, cost) or logistic regression (binary outcomes) with cluster-robust standard errors (clustered by hospital). Interaction *p*-value tests the insurance × IDU interaction term in each model. Reference category: insured (Medicare, Medicaid, or private insurance). ^†^ IRR = incidence rate ratio from Gamma regression with log link; a value of 1.09 indicates approximately 9% longer LOS among uninsured patients. ^‡^ Exponentiated Gamma regression coefficient; a value of 0.90 indicates approximately 10% lower total costs among uninsured patients (Figure 3).

In a sensitivity analysis stratifying the insured category into Medicare/Medicaid versus private insurance (Table 5), a consistent gradient emerged across all outcomes: Medicare/Medicaid beneficiaries had significantly worse outcomes than privately insured patients but better outcomes than uninsured patients. Compared with privately insured patients, uninsured patients had 4.5-fold higher adjusted odds of AMA discharge (aOR 4.48, 95% CI 3.88–5.16) and 38% higher adjusted in-hospital mortality (aOR 1.38, 95% CI 1.22–1.56), while Medicare/Medicaid patients had intermediate estimates (aOR 2.28 for AMA; aOR 1.10 for mortality). This gradient persisted across all LOS and cost outcomes.

To address the clinical context of in-hospital deaths, we compared the prevalence of complications and procedures recorded during terminal hospitalizations between insured and uninsured decedents (Table 6). The terminal clinical profiles were broadly similar between groups. Rates of sepsis or septic shock (20.5% vs. 20.9%, *p* = 0.844), acute kidney injury (21.9% vs. 22.6%, *p* = 0.746), cardiac arrest (3.6% vs. 4.5%, *p* = 0.354), and valve or cardiac surgery (49.4% vs. 48.0%, *p* = 0.547) did not differ significantly. Acute respiratory failure was somewhat more common among uninsured decedents (25.2% vs. 21.8%, *p* = 0.091), as were stroke or cerebrovascular events (8.9% vs. 7.3%, *p* = 0.192), though neither reached statistical significance. The only significant difference was a higher prevalence of congestive heart failure among insured decedents (23.9% vs. 16.9%, *p* < 0.001), consistent with their older age and greater cardiovascular comorbidity burden.

## 4. Discussion

### 4.1. Principal Findings

In this national cross-sectional analysis of 87,211 weighted IE hospitalizations from 2016 to 2019, uninsured adults experienced substantially longer hospital stays than insured adults, with particularly large disparities at the extreme tail: uninsured patients had 46% higher adjusted odds of remaining hospitalized beyond 28 days (aOR 1.46, 95% CI 1.30–1.65). AMA discharge showed the strongest association, with uninsured patients having 3.5-fold higher adjusted odds of leaving before completing therapy (aOR 3.51, 95% CI 3.10–3.97). These associations persisted after multivariable adjustment for age, sex, race/ethnicity, income, comorbidity burden, IDU, and hospital characteristics, and the insurance × IDU interaction confirmed that AMA disparities were most pronounced among non-IDU patients.

A particularly notable finding emerged for in-hospital mortality. In the unadjusted analysis, mortality appeared similar between groups (9.0% vs. 9.4%, *p* = 0.32). However, after adjusting for the substantially younger age and lower comorbidity burden of the uninsured cohort, uninsured status was associated with 25% higher odds of in-hospital death (aOR 1.25, 95% CI 1.10–1.43). The unadjusted equivalence had masked a disparity: uninsured patients, given their favorable age and comorbidity profile, should have had substantially lower mortality than insured patients—but they did not. This finding strengthens the interpretation that uninsured IE patients face meaningful barriers to optimal care that extend beyond discharge planning to encompass in-hospital management itself.

Potential mechanisms by which insurance status may affect in-hospital mortality include differential access to subspecialty consultation. Uninsured patients may have reduced access to cardiothoracic surgery evaluation, infectious disease consultation, and advanced echocardiographic monitoring—all of which are integral to the Endocarditis Team model of care, increasingly recognized as the standard for IE management. Differential diagnostic intensity, including rates of repeat imaging to monitor for embolic complications or vegetation progression, may also contribute. These mechanisms are not directly measurable in administrative data but represent plausible pathways deserving prospective investigation.

The comparison of terminal clinical profiles (Table 6) provides further context for the adjusted mortality disparity. Because the prevalence of acute complications among decedents was broadly similar between insured and uninsured patients, the higher adjusted mortality in the uninsured group does not appear to reflect a distinct or more lethal disease pattern. This pattern is consistent with the interpretation that differences in the processes of care, rather than differences in the intrinsic severity of terminal illness, contribute to the observed mortality gap—though, as noted, the National Inpatient Sample does not capture a formal cause of death, and this interpretation remains inferential.

### 4.2. Mechanism: OPAT and Post-Acute Care Access

Our findings align with the logistical realities of IE management. The standard 4- to 6-week course of intravenous antibiotics required for IE creates a unique dependency on post-acute care infrastructure [2,3]. For insured patients who achieve clinical stability, transition to OPAT—at home with nursing support or in an SNF—allows completion of antibiotic therapy outside the acute care setting [8,9,10,11,12]. This transition typically occurs within the first 1 to 2 weeks of hospitalization once patients have cleared bacteremia, demonstrated hemodynamic stability, and met other established criteria for OPAT candidacy [14].

For uninsured patients, this transition pathway is often inaccessible. Home OPAT requires nursing visits, infusion supplies, and laboratory monitoring—services that are rarely available without insurance coverage or substantial out-of-pocket payment. Similarly, SNFs may decline admission for patients without a funding mechanism to cover the stay. The result is that clinically stable uninsured patients may remain hospitalized solely because no safe discharge destination exists—a phenomenon sometimes termed “social admission” or “disposition-related hospitalization.” The 46% higher adjusted odds of extreme prolonged hospitalization (>28 days) is particularly notable: a hospitalization exceeding 4 weeks suggests persistent, often intractable barriers to discharge. From a patient-centered perspective, these stays represent more than a month separated from home, family, and work; from a health-systems perspective, they represent inefficient use of acute care beds and increased exposure to nosocomial complications, including hospital-acquired infections and deconditioning.

### 4.3. Discharge Against Medical Advice

The 3.5-fold higher adjusted odds of AMA discharge among uninsured patients is particularly important because IE requires uninterrupted intravenous therapy to prevent relapse and complications, and premature departure likely contributes to adverse long-term outcomes that are not captured in this inpatient analysis. The AMA disparity may represent a rational response by patients to an untenable situation: facing prolonged hospitalization without a clear discharge pathway, some patients ultimately choose to leave before completing therapy. The significant insurance × IDU interaction (interaction aOR 0.44, *p* < 0.001) indicates that the insurance effect on AMA was strongest among non-IDU patients, consistent with the six-fold unadjusted disparity in that subgroup (15.2% vs. 2.5%). The IDU population has baseline elevated AMA rates attributable to factors including active addiction, withdrawal symptoms, and stigma-related experiences in healthcare settings [37,38,39,40]. The AMA rates observed here among IDU patients (22.4% insured, 33.4% uninsured) are consistent with those reported in prior single-center and regional studies of IDU-associated IE, lending external validity to the present dataset. Our findings indicate that lack of insurance compounds these existing challenges while also creating substantial and independent barriers for non-IDU patients who would otherwise have straightforward discharge trajectories [41,42].

### 4.4. Alternative Explanations

Several alternative explanations warrant consideration. First, residual confounding by unmeasured disease severity—causative organism, vegetation size, valve involvement, or hemodynamic complications not captured in administrative data—could in principle contribute to longer stays among uninsured patients. However, the persistence of LOS and AMA associations after multivariable adjustment for age, comorbidity burden, and IDU argues against measured confounders as the dominant explanation, while the adjusted mortality finding (aOR 1.25) suggests that if unmeasured severity confounding exists, it operates against uninsured patients rather than explaining their longer stays as a marker of clinical need. Second, social determinants beyond insurance—unstable housing, lack of caregiver support, food insecurity—could prolong admissions independently of payer status; however, these factors are themselves correlated with being uninsured and would, if anything, reinforce the policy implications of our findings. Third, in the IDU subgroup, AMA discharge could reflect addiction-related factors, including withdrawal management, stigma in healthcare encounters, and ongoing substance use rather than purely insurance-driven dynamics [37,38,39,40]. The significant insurance × IDU interaction, with a stronger insurance–AMA association among non-IDU patients, supports the conclusion that insurance-related barriers operate independently of addiction. Fourth, differential receipt of cardiac surgery (44.0% vs. 39.7%) might also influence LOS, though the direction—lower surgical rates in the uninsured—is opposite to what would be expected if surgery were driving the LOS gap.

The insured category in this analysis encompasses meaningful heterogeneity. Medicare and Medicaid beneficiaries face substantially different OPAT and skilled nursing facility access than privately insured patients: Medicaid reimbursement for home infusion services is inconsistent across states, and Medicare’s home health benefit imposes eligibility criteria that can be difficult to meet. To characterize this gradient, we performed a sensitivity analysis stratifying the insured group into Medicare/Medicaid versus private insurance (Table 5). Compared with privately insured patients, Medicare/Medicaid beneficiaries had intermediate but significantly worse outcomes across every domain examined: 14% higher adjusted odds of prolonged LOS > 14 days (aOR 1.14, 95% CI 1.09–1.20), 26% higher odds of LOS > 28 days (aOR 1.26, 95% CI 1.17–1.36), more than two-fold higher odds of AMA discharge (aOR 2.28, 95% CI 2.03–2.57), and 10% higher adjusted in-hospital mortality (aOR 1.10, 95% CI 1.03–1.18). Uninsured patients showed the largest disparities in every domain. The consistent gradient—private < Medicare/Medicaid < uninsured—confirms that insurance-related barriers operate along a spectrum, and indicates that the binary classification used in our primary analysis, while conservative, likely understated the magnitude of disparity between the most and least advantaged groups.

### 4.5. Limitations

This study has several important limitations. First, although multivariable adjustment was performed, the analysis remains cross-sectional and observational; the associations reported should not be interpreted as causal estimates. Residual confounding by unmeasured factors—including disease severity markers not available in the NIS (causative organism, vegetation characteristics, hemodynamic parameters)—cannot be excluded. Second, we could not directly assess OPAT eligibility or specific reasons for discharge delay; OPAT-related mechanisms are inferred from population-level patterns rather than measured directly [27]. Third, the NIS captures only inpatient admissions, and we could not assess 30-day readmissions, post-discharge complications, or long-term mortality, which are likely particularly relevant for AMA patients. No post-discharge surveillance is available in the NIS; the true clinical impact of the 3.5-fold higher AMA rate among uninsured patients—including IE relapse, need for valve surgery, and long-term mortality—therefore cannot be quantified from this dataset and represents an important direction for future linked-data studies. Fourth, insurance status reflects the primary expected payer at discharge; in-hospital Medicaid enrollment would lead some patients with effectively no admission-time coverage to be classified as insured, biasing our findings toward the null.

Fifth, ICD-10–based identification of IE is subject to misclassification, although prior validation suggests positive predictive values exceeding 80%, and we cannot distinguish definite from possible IE per Duke criteria [28,29,43]. Sixth, the study period (2016–2019) ends prior to the COVID-19 pandemic, which substantially altered IE epidemiology and post-acute care access; results may not generalize to the post-pandemic era. Seventh, we did not capture homelessness or housing instability separately, although these factors are likely correlated with being uninsured. Eighth, the LOS thresholds we selected (>14 and >28 days) are clinically motivated but not formally validated; sensitivity analyses using alternative thresholds were not performed. Finally, while NIS discharge weights and hospital-level clustering were incorporated into the multivariable models, full stratification using NIS_STRATUM was not applied in the regression estimation; this limitation is expected to produce conservative (slightly overestimated) standard errors and does not affect point estimates. In practical terms, the confidence intervals reported here are slightly wider than they would be with full survey specification—meaning the associations we report are held to a higher bar for statistical significance than strictly necessary, and our findings are therefore conservative rather than inflated.

### 4.6. Implications

Our findings have several implications. Clinically, they identify uninsured IE patients as a high-risk population for prolonged hospitalization, AMA discharge, and—after adjustment—higher in-hospital mortality, all of which carry meaningful adverse consequences. The adjusted mortality finding is particularly concerning: it suggests that insurance-related barriers affect not only discharge planning but potentially the quality or intensity of in-hospital care itself. These results underscore the value of early multidisciplinary discharge planning that explicitly addresses payer status and post-acute care access. From a policy standpoint, although the Affordable Care Act’s Medicaid expansion has reduced the ranks of the uninsured [23,24], significant gaps remain in non-expansion states, and even among Medicaid beneficiaries, reimbursement and network adequacy issues can limit OPAT and SNF availability.

**Figure 4:**
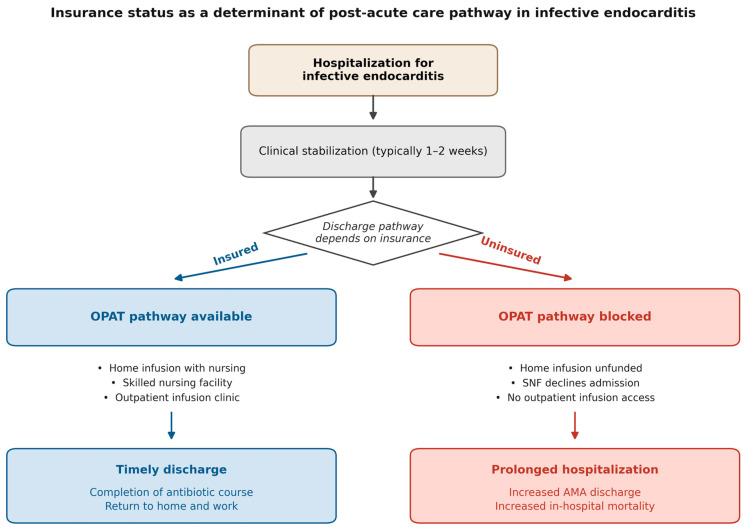
Conceptual model illustrating how insurance status determines the post-acute care pathway in infective endocarditis. For insured patients, OPAT and post-acute facility access enable timely discharge; for uninsured patients, blocked OPAT access leads to prolonged hospitalization, higher AMA discharge rates, and worse adjusted outcomes.

Alternative care models offer pathways to address these disparities. Hospital-based OPAT clinics, where patients return daily for antibiotic administration, can provide a middle ground between full hospitalization and home-based OPAT for patients without adequate home support [15]. Some institutions have developed respite or medical-shelter programs that provide housing and nursing support for unstably housed patients completing antibiotic courses. Finally, the growing evidence base supporting partial oral antibiotic therapy for selected IE cases, as demonstrated in the POET trial and its long-term follow-up [44,45,46], offers a particularly promising avenue for uninsured patients specifically. Because partial oral step-down therapy eliminates the need for home infusion infrastructure, nursing visits, and maintenance of intravenous access, it bypasses the primary insurance-dependent bottleneck that drives prolonged hospitalization in this population. If oral step-down protocols were applied to eligible uninsured IE patients, the discharge barrier that currently accounts for weeks of excess hospitalization could be substantially reduced without requiring insurance reform. Although not yet standard of care for all IE presentations, expanding the clinical adoption of partial oral regimens represents a near-term, actionable intervention for this high-risk group (Figure 4).

## 5. Conclusions

In a national cross-sectional analysis of adult IE hospitalizations, uninsured patients experienced significantly longer hospital stays, markedly higher rates of discharge against medical advice, and—after multivariable adjustment for age, comorbidity, and IDU—higher in-hospital mortality than insured patients. The persistence of these associations after adjustment and the finding that unadjusted mortality equivalence masked an adjusted disparity are consistent with nonclinical barriers to discharge and potentially to optimal in-hospital care—particularly limited OPAT and post-acute care access. The results identify uninsured IE patients as a population for whom policy reforms, expanded safety-net programs, and alternative care models—including hospital-based OPAT clinics and partial oral therapy approaches—could meaningfully reduce institutional confinement and the risk of premature treatment discontinuation.

## Figures and Tables

**Table 1:** Baseline characteristics of patients hospitalized with infective endocarditis by insurance status (National Inpatient Sample 2016–2019).

Characteristic	Insured (*n* = 81,667)	Uninsured (*n* = 5544)	*p*-Value
**Demographics**			
Age, years, mean ± SD	59.4 ± 20.1	40.1 ± 13.2	<0.001
Male sex, %	55.7	54.5	0.09
Race/ethnicity, %			<0.001
White	70.6	75.3	
Black	12.8	9.1	
Hispanic	8.1	10.0	
Other	8.4	5.7	
Lowest income quartile, %	31.8	43.4	<0.001
**Comorbidities**			
Injection drug use, %	15.5	38.7	<0.001
Diabetes mellitus, %	31.0	12.8	<0.001
Chronic kidney disease, %	31.4	9.1	<0.001
Heart failure, %	42.2	20.6	<0.001
HIV/AIDS, %	1.4	1.9	0.003
**Clinical Features and Hospital Characteristics**			
Cardiac surgery, %	44.0	39.7	<0.001
Teaching hospital, %	7.6	6.7	0.02
Urban hospital, %	92.4	93.3	0.01

Abbreviations: SD, standard deviation; HIV, human immunodeficiency virus; AIDS, acquired immunodeficiency syndrome. All percentages are weighted estimates. *p*-values from Pearson chi-square tests for categorical variables and Student’s *t*-tests for continuous variables, with NIS survey design accounted for in variance estimation.

**Table 2:** Hospitalization outcomes by insurance status.

Outcome	Insured (*n* = 81,667)	Uninsured (*n* = 5544)	*p*-Value
**Length of Stay**			
Mean ± SD, days	12.4 ± 14.8	15.5 ± 17.4	<0.001
Median [IQR], days	8.0 [4.0–15.0]	9.0 [4.0–22.0]	<0.001
>14 days, %	26.6	35.8	<0.001
>28 days, %	9.2	18.5	<0.001
**Discharge Disposition**			
In-hospital mortality, %	9.4	9.0	0.32
Routine discharge home, %	24.3	37.7	<0.001
Discharge against medical advice, %	5.5	22.2	<0.001
**Cost**			
Mean hospitalization cost, USD	44,393	41,061	0.02

Abbreviations: SD, standard deviation; IQR, interquartile range. Costs were estimated by multiplying total charges by hospital- and year-specific cost-to-charge ratios provided by the Healthcare Cost and Utilization Project.

**Table 3:** Outcomes stratified by injection drug use status.

Panel A. Patients without injection drug use (*n* = 72,432).			
**Characteristic/Outcome**	**Insured (*n* = 69,034)**	**Uninsured (*n* = 3398)**	***p*** **-Value**
Age, years, mean ± SD	63.4 ± 18.6	43.7 ± 14.3	<0.001
Male sex, %	57.4	61.0	<0.001
Heart failure, %	46.5	25.9	<0.001
Length of stay, mean ± SD, days	11.8 ± 14.4	14.4 ± 17.2	<0.001
LOS > 14 days, %	24.5	33.1	<0.001
LOS > 28 days, %	7.5	15.3	<0.001
In-hospital mortality, %	10.1	10.5	0.46
Routine discharge home, %	23.8	39.8	<0.001
Discharge against medical advice, %	2.5	15.2	<0.001
Panel B. Patients with injection drug use (*n* = 14,779).			
**Characteristic/Outcome**	**Insured (*n* = 12,633)**	**Uninsured (*n* = 2146)**	***p*** **-Value**
Age, years, mean ± SD	37.6 ± 12.9	34.6 ± 8.5	<0.001
Male sex, %	46.3	44.1	0.06
Heart failure, %	18.7	12.3	<0.001
Length of stay, mean ± SD, days	16.0 ± 16.3	17.2 ± 17.5	0.003
LOS > 14 days, %	37.9	40.2	0.04
LOS > 28 days, %	18.1	23.5	<0.001
In-hospital mortality, %	5.5	6.5	0.08
Routine discharge home, %	27.2	34.4	<0.001
Discharge against medical advice, %	22.4	33.4	<0.001

Abbreviations: SD, standard deviation; LOS, length of stay. *p*-values compare insured versus uninsured patients within each panel.

**Table 5:** Multivariable-adjusted associations between insurance category and hospitalization outcomes (three-category sensitivity analysis).

Outcome/Comparison	Adjusted Estimate	95% CI	*p*-Value
**LOS, days (IRR)**			
Medicare/Medicaid vs. private	1.09	1.06–1.11	<0.001
Uninsured vs. private	1.16	1.11–1.21	<0.001
**LOS > 14 days (aOR)**			
Medicare/Medicaid vs. private	1.14	1.09–1.20	<0.001
Uninsured vs. private	1.25	1.16–1.36	<0.001
**LOS > 28 days (aOR)**			
Medicare/Medicaid vs. private	1.26	1.17–1.36	<0.001
Uninsured vs. private	1.64	1.47–1.84	<0.001
**Discharge against medical advice (aOR)**			
Medicare/Medicaid vs. private	2.28	2.03–2.57	<0.001
Uninsured vs. private	4.48	3.88–5.16	<0.001
**In-hospital mortality (aOR)**			
Medicare/Medicaid vs. private	1.10	1.03–1.18	0.006
Uninsured vs. private	1.38	1.22–1.56	<0.001
**Total cost (exp. coeff.)**			
Medicare/Medicaid vs. private	0.95	0.90–1.00	0.030
Uninsured vs. private	0.87	0.80–0.94	0.001

All models adjusted for age, sex, race/ethnicity, median household income quartile, injection drug use (IDU), Elixhauser Comorbidity Index (modified), hospital bed size, teaching status, and region. Reference category: private insurance. The insurance × IDU interaction term included in the primary analysis (Table 4) was omitted for this sensitivity analysis. IRR = incidence rate ratio from Gamma regression with log link; aOR = adjusted odds ratio from logistic regression. Exponentiated coefficient for total cost from Gamma regression with log link.

**Table 6:** Prevalence of complications and procedures among patients who died in-hospital, by insurance status (terminal clinical profile).

Condition/Procedure	Insured (%)	Uninsured (%)	*p*-Value
Sepsis/septic shock	20.5	20.9	0.844
Acute kidney injury	21.9	22.6	0.746
Cardiac arrest	3.6	4.5	0.354
Stroke/cerebrovascular event	7.3	8.9	0.192
Acute respiratory failure	21.8	25.2	0.091
Congestive heart failure	23.9	16.9	<0.001
Valve/cardiac surgery	49.4	48.0	0.547

Analysis restricted to hospitalizations ending in in-hospital death. Percentages are weighted using NIS discharge weights; *p*-values from Pearson chi-square tests. Conditions and procedures were identified from all-listed ICD-10-CM diagnoses and ICD-10-PCS procedure codes and are not mutually exclusive; column totals therefore exceed 100%. The National Inpatient Sample does not record a formal cause of death, and these variables represent the clinical context surrounding death rather than an adjudicated cause. Unweighted event counts ranged from 22 to 3751 per cell, all exceeding HCUP reporting thresholds.

## Data Availability

The data are publicly available from the Healthcare Cost and Utilization Project (HCUP) at https://www.hcup-us.ahrq.gov/nisoverview.jsp (accessed on 3 February 2026), subject to HCUP data use terms.
